# Evaluation of the Accuracy of Capillary Hydroxybutyrate Measurement Compared with Other Measurements in the Diagnosis of Diabetic Ketoacidosis: A Systematic Review

**DOI:** 10.3390/ijerph13090837

**Published:** 2016-08-23

**Authors:** Joanne Brooke, Marlon Stiell, Omorogieva Ojo

**Affiliations:** 1 College of Nursing, Midwifery and Healthcare, University of West London, Boston Manor Road Brentford, Middlesex TW8 9GA, UK; 2 Faculty of Education and Health, University of Greenwich, Avery Hill Campus, London SE9 2UG, UK; m.stiell@greenwich.ac.uk (M.S.); o.ojo@greenwich.ac.uk (O.O.)

**Keywords:** diabetes ketoacidosis, type 1 diabetes, type 2 diabetes, emergency department, point of care testing, ketones

## Abstract

A complication of diabetes is diabetic ketoacidosis (DKA), which if left untreated is a life threatening condition. Prompt and accurate diagnosis of DKA is required for the commencement of life saving interventions. Measurements of ketone bodies in DKA have usually been through nitroprusside urine acetoacetate testing. The aim of this systematic review was to examine whether capillary β-hydroxybutyrate (β-OHB) testing is more accurate compared to other diagnostic methods of DKA. The following electronic databases were searched: EBSCO Host, MEDLINE, PSYCHInfo, CINAHL and Science Direct for publications from 1 January 2005 and up to and including 1 January 2016. Inclusion criteria were: Adults 18 years and over and known type 1 or type 2 diabetes. Retrospective and prospective observation studies were included. A total of nine studies met the inclusion criteria. Capillary β-OHB was found to have high sensitivity, specificity, positive predictive value and negative predictive value in identifying DKA compared to urinary ketone testing.

## 1. Introduction

Diabetic Ketoacidosis (DKA) is a life threatening complication of diabetes, which is characterized by a triad of hyperglycaemia, ketosis and metabolic acidosis [1,2,3]. It results from absolute or relative insulin deficiency leading to the failure of glucose to enter the cells for the normal metabolic pathways. Therefore, the lack of insulin secretion triggers the release of counter regulatory hormones including cathecholamines, glucagon and cortisol. The imbalance in hormone leads to hepatic gluconeogenesis and glycogenolysis, resulting in severe hyperyglycemia [1]. Although lipolysis is a normal biochemical process, it becomes unregulated in DKA. The process of ketone production in DKA involves the formation of serum free fatty acids, which are used for the production of large quantities of ketone bodies (acetoacetate, β-hydroxybutyrate (β-OHB), and acetone), and consequent metabolic acidosis [1,3]. While the liver is the site of ketone formation, the first ketone body produced is acetoacetic acid, which is reduced to β-OHB or acetone [2]. Therefore, the β-OHB to acetoacetate ratio, which is 1:1 in its normal state, is usually elevated to at least 3:1 in diabetic ketoacidosis at presentation [4] and may be as much as 10:1 [5]. β-OHB levels are the most abundant in the blood in DKA, representing 75% of total ketones formed [6]. The blood β-OHB levels below 0.6 mmol/L are regarded as normal, while levels over 1 mmol/L represent hyperketonemia, and levels in excess of 3 mmol/L indicate ketoacidosis [6]. 

The characteristic features of DKA, such as osmotic diuresis resulting from hyperglycaemia, may lead to fluid depletion, dehydration and electrolyte imbalance [1]. According to Klocker et al. [7] and Mackay et al. [8], DKA may occur during episodes of infection, stress, insulin omission and interruption of insulin by an insulin pump. 

Although DKA is most common in patients with Type 1 diabetes, it may also occur in those with Type 2 diabetes. It accounts for 8%–29% of all hospital admissions of patients with diabetes as the primary diagnosis with average mortality rates of 5%–10%, and as high as 15% in the elderly population [9]. It should be emphasised that patients require prompt recognition, treatment and monitoring from health care professional. 

In patients with DKA, it is essential to take the patient’s history and review the presenting signs and symptoms which may include excessive thirst, feeling tired and lethargic, blurred vision, abdominal pain, nausea, vomiting, and changes in respiration [10]. In addition, physical examination should be conducted and blood and urine tests should be carried out in order to provide preliminary diagnosis of DKA [11]. The measurement of ketone bodies in either serum or urine is often undertaken by dipsticks, which rely on the nitroprusside test that changes the colour of the stick to purple–violet [12]. According to Dhatariya the urine ketone stick test such as Ketostix by Bayer Diabetes, Berkshire, UK, gives a semi-quantitative measurement of acetoacetate and not β-OHB which is the most predominant ketone body in DKA [13]. In addition, there are other limitations with the use of the dipsticks for carrying out these tests. For example, the use of urinary dipstick ketone test in DKA diagnosis and management may be unreliable because β-OHB is converted to acetoacetate during treatment of DKA [4]. In addition, urine sample can take time or may even be impossible in seriously ill patients in the emergency setting [14,15]. On the other hand, Misra and Oliver, and Katsilambros et al. noted that measurement of ketones from samples of serum, plasma, or whole blood may involve using laboratory based analysers or from finger prick samples using capillary blood [12,16]. These samples can be analysed for β-OHB using different assays, such as spectrophotometry, colorimetric, gas chromatography, capillary electrophoresis and enzymatic, although the enzymatic method appear to be the most widely used [16]. The enzymatic method for β-OHB measurement described by Noyes et al. [5] involved the use of Hitachi 911 analyser (Roche Diagnostic, London, UK) in which β-OHB dehydrogenase catalyses the conversion of the D isomer of β-OHB and nicotine amide dinucleotide (NAD) to acetoacetate and nicotine amide dinucleotide hydrogen (NADH) at pH 8.5.

However, laboratory measurement of β-OHB is not routinely available and it takes too long to be of practical use in the emergency diagnosis and in the management of DKA in home settings [17,18].

Therefore, the use of new meters, which can measure β-OHB in capillary blood, are now available although the accuracy of these new meters have not been well tested against other methods. In the UK, two meters used to measure capillary blood ketone concentrations are the GlucoMen LX Plus by Menarini Diagnostics and Freestyle Optium Neo by Abbot Diabetes care [13]. In contrast to the laboratory methods of detecting β-OHB [5] reported in their study that a 5-µL finger prick capillary blood sample is required for the measurement of capillary β-OHB and results are usually displayed within 30 s. The meter analytical range was 0–6 mmol/L, with results >6 mmol/L being displayed as “HI”. The American Diabetes Association, since 2004 [19], has recommended blood β-OHB for diagnosis and treatment of ketoacidosis. 

In a previous systematic review which examined the use of blood β-OHB and urine acetoacetate testing for the prevention of and management of ketoacidosis, Klocker et al. [7] focused only on patients with Type 1 diabetes and with age of subjects below 22 years. However, the focus of the current review was adults over the age of 18 with Type 1 or Type 2 diabetes.

Based on the above, the aim of this review is to evaluate whether capillary β-OHB measurement is more accurate compared to other measurements in the diagnosis and management in patients presenting with diabetic ketoacidosis.

The objective is as follows:

- To examine the use of capillary β-OHB compared to urinary and laboratory methods used in the detection and management of diabetic ketoacidosis.

The research question is:

- Is capillary β-OHB as accurate in measuring ketones compared with urinary and laboratory methods in patients with diabetic ketoacidosis?

## 2. Methods

The literature search strategy for this review relied on previously published guidelines for systematic reviews [20,21]. A number of databases including EBSCO host, encompassing Academic search premier, Medline, Psychology and Behavioural sciences collection, PSYCINFO, SPORTDISCUSS and Cumulative Index to Nursing and Allied Health Literature (CINAHL) Plus and Science Direct were accessed for relevant evidence. References of articles were also checked in order to find useful evidence for the review. A range of search terms were used and these included medical subject headings (MeSH), such as “diabetic ketoacidosis” (refer to Table 1).

In addition, “Bolean” operators allowing the combination of search terms such as “diabetic ketoacidosis” and “blood ketones”; “diabetic ketoacidosis” and “capillary ketones”; “diabetic ketoacidosis” and “capillary β-hydroxybutyrate”; “diabetic ketoacidosis” and “ketone, urine” were used.

This review involved splitting the research question into its component parts, namely; Population (P), Intervention (I), Comparative interventions (C) and Outcomes (O) based on the PICO framework [20]. In this regard, the population of interest was patients with diabetes presenting with diabetic ketoacidosis, while the intervention was the capillary blood ketone measurement and the comparative interventions included urinary ketone testing. The outcomes of interests were the ketone measurements obtained from these procedures and the accuracy in predicting a diagnosis of diabetic ketoacidosis.

## 3. Inclusion Criteria

The selection of articles for this review was based on a number of set criteria. Firstly, the articles selected were only those published between 2005 and 2016. This was to enable enough period of time to have sufficient articles for the review.

Primary research articles based on original and/or new data were selected. In addition, studies involving secondary data analysis based on retrospective and prospective studies were also included. Articles selected were those written in English and included studies with participants aged 18 years or over.

## 4. Exclusion Criteria

Studies involving children and animals were excluded from the review. In addition, duplicate citations were identified and excluded. All studies that did not meet the criteria for inclusion were excluded from the review.

## 5. Quality Assurance

In order to ensure the quality of this review, three researchers were independently involved in the searches based on an agreed search criteria including search terms. Results of the searches were then harmonised and evaluated. The evaluation process involved assessing the quality of the research articles and this was based on previously published reviews, based on the experience of the researchers and on the Scottish Intercollegiate Guidelines Network (SIGN) checklist for critical appraisal [7,22].

## 6. Results

The search identified nine studies that met the inclusion criteria of which: one compared capillary β-OHB with blood glucose levels ≥10.0 mmols/L [4]; two compared capillary β-OHB with standard measures (arterial blood gases, anion gap and carbon dioxide) of DKA [23,24], although one did not include urine ketones [23]; one compared capillary β-OHB with the manual enzymatic method [25]; five compared capillary β-OHB with urine ketones in detecting DKA [6,14,25,26,27] (refer to Table 2).

The studies included a total of 2019 participants, with a range of 19–529. Studies were completed in Australia [9], Banglaesh [27], France [14], Greece [25], Singapore [24], South Africa [28], Thailand [6], and USA [23,26].

Due to inconsistencies in methodologies, definitions and cut off levels of capillary β-OHB and blood glucose, and biological measures reported across the studies, a meta-analysis of the data was not possible. 

Of the five studies that compared capillary β-OHB with urine ketones, capillary β-OHB was found to be equally as sensitive as urine ketones for detecting DKA at the level of >1.5 mmol/L, however, capillary β-OHB was more specific than urine ketones (78.6% versus 35.1%) in the ED [26], whilst in a small sample of 19 participants capillary β-OHB at the level of >3.0 mmols/L had a lower sensitivity than urine ketones (90% versus 95%) and equal specificity of 100% [6]. In a sample of 50 participants capillary β-OHB had a higher sensitivity, specificity and positive predictive value than urine ketones (99.87% versus 89.89%, 92.89% versus 52.78%, and 92.89% versus 41.87%) in detecting DKA in ED [25]. However, in a sample of 121 participants capillary β-OHB at the level of >3.0 mmols/L as the reference method, sensitivity of urine ketones was reported at 32.6% and specificity at 93.7%, with a positive predictive value of 73.68% and negative predictive value of 71.84% [27]. The relative risk of DKA of capillary β-OHB ≥ 3.0 mmols/L was higher than the measurement of +++ urine ketones 74 (95% CI: 48–88) versus 31 (95% CI: 18–45), low values of capillary β-OHB and urine ketones demonstrated a good correlation, but a poor correlation between high values was found [14]. 

Of the two studies that compared capillary β-OHB with standard measures of DKA, capillary β-OHB value of >1.5 mmols/L had a sensitivity 100% and 98%, specificity of 93.3% and 85%, positive predictive value of 50%, and a negative predictive value of 100%, a positive likelihood ratio of 6.7 (95% CI: 4.22–10.78) and a negative likelihood ratio of 0.021 (95% CI: 0.003–0.144) of detecting hyperketonaemia [23,24]. Whereas a capillary β-OHB value of >3.5 mmols/L had a sensitivity 100%, specificity of 100%, positive predictive value 100%, and a negative predictive value of 100% [24] of detecting DKA.

Two studies found a modest and high correlation between blood glucose and capillary β-OHB (*r* = 29, *p* = 0.02 and *r* = 0.3, *p* < 0.001) [9,23]. 

## 7. Discussion

Based on the results of this review there is evidence of differences in terms of sensitivity, specificity, positive predictive value and negative predictive value between capillary β-OHB testing and urinary ketone testing. The differences observed could be due to a number of factors. Firstly, urinary ketone test involves a reaction between nitroprusside and acetoacetate which leads to a colour change on the test strip that is then compared subjectively against a reference standard [8]. This procedure is time dependent and it is therefore advised to disregard the colour changes after two minutes. Furthermore, the nitroprusside test does not detect β-OHB, while sulphydryl drugs, such as captopril could cause false–positive results and exposure of the test strips to air may lead to false–negative outcomes [8].

In contrast, the meter method for testing capillary blood ketone measures mainly β-OHB, which is the predominant ketone in DKA and usually oxidised to acetoacetate as the ketosis resolves [5,13]. According to Yu et al. [29], due to the fact that β-OHB is metabolised to acetoacetate during treatment, the ketone levels in urine often rise despite decreasing concentration of blood β-OHB. Therefore, the urine ketone testing may give misleading information about the degree of ketosis and/or may suggest that the DKA is not resolving [5]. Dhatariya [13] argued that urine ketone test measures the average concentration in the urine that is held in the bladder since the last elimination and this may have implications for patients with DKA who are often dehydrated.

The high correlation between capillary β-OHB levels and the manual enzymatic method is in line with previous report, which showed good correlation between these meters and enzymatic spectrophotometric assays [16]. 

It was also clear from this review that β-OHB compared well with other measures of DKA such as carbon dioxide and blood glucose and is in tandem with previous reports. According Klocker et al. [7], β-OHB is the predominant ketone body in DKA and correlates well with acidosis. In addition, Federici and Benedetti [18] observed that different authors have indicated that capillary β-OHB levels showed a relationship with the level of metabolic control as defined by glucose and HBA1_c_. For example, it has been demonstrated that capillary β-OHB levels are closely related with other markers of DKA including free fatty acid levels and blood pH [18].

The importance of capillary β-OHB testing was highlighted for a number of reasons; in one study over half of participants were not able to produce a urine sample in ED [24], one study identified three cases of DKA mortality where a negative test for urine ketones had been recorded, and urine ketones did not demonstrate or correlate with the severity of capillary β-OHB, as low urine ketones in participant was matched with a β-OHB measurement of 4.1 mmols/L. 

Despite the advantages of the use of Point of Care meters for measuring capillary β-OHB, there are limitations. According to Dhatariya [13], there is evidence of poor performance of Point of Care meters at high levels of ketone concentration. This position is supported by Yu et al. [29] who demonstrated that the use of Abbott meter for the measurement of β-OHB correlates well with the reference laboratory method (Stanbio Assay) up to 3 mmol/L, and becomes discrepant after that value. The authors further observed that while the Abbott meter may be useful for the diagnosis of DKA, it may present challenges to clinicians in the management of patients with DKA who have β-OHB values greater than 5 mmol/L (reference method) in hospital settings. 

## 8. Limitations of the Review

The different methods and designs of the studies included in this review made comparison of the studies difficult. In addition, these studies were conducted in various countries around the world with different research standards and quality.

## 9. Conclusions

The use of capillary β-OHB testing is more accurate than urinary ketone testing and compares well with the enzymatic method. This approach will be useful in early identification of DKA.

Capillary β-OHB testing is a simple tool that is an accurate, precise method of early identification of DKA in the Emergency Department and reduces unnecessary delays in diagnosis of DKA. Capillary β-OHB and urine ketones had high sensitivity, but urine ketones had low specificity in identifying DKA in the ED, with an error of almost 30% of false positive results. Capillary β-OHB testing is more accurate than urine ketones, as accurate as manual enzymatic method, and highly correlated with a clinical diagnosis of DKA.

## Figures and Tables

**Table 1:** Literature search strategy.

Key Words	Search Engine	Hits	Search Engine	Hits
Diabetic ketoacidosis	EBSCO host *	5583	Science Direct	2299
“Diabetic ketoacidosis“ and “beta hydroxybutyrate“	EBSCO host	109	Science Direct	159
“Diabetic ketoacidosis“and “acetoacetate“	EBSCO host	43	Science Direct	113
Early detection of ketones	EBSCO host	3	Science Direct	12
“Diabetic ketoacidosis“ and “point of care testing“	EBSCO host	38	Science Direct	663
“Diabetic ketoacidosis“ and “early detection of ketones“	EBSCO host	1	Science Direct	83
“Diabetic ketoacidosis“ and “measurement of blood ketones“	EBSCO host	12	Science Direct	185
“Diabetic ketoacidosis“ and “enzymatic method“	EBSCO host	9	Science Direct	171
“Diabetic ketoacidosis“ and “blood ketones“	EBSCO host	85	Science Direct	1636
“Diabetic ketoacidosis“ and “urine ketones“	EBSCO host	81	Science Direct	1096
“Diabetic ketoacidosis“ and “capillary ketones“	EBSCO host	10	Science Direct	433
“Diabetic ketoacidosis“ and “serum beta hydroxybutyrate“	EBSCO host	8	Science Direct	401
“Diabetic ketoacidosis“ and “laboratory analysis“	EBSCO host	9	Science Direct	2059

* EBSCO host included: Academic Search Premier, Medline, Psychology and Behavioural Sciences Collection, PSYCHOINFO, SPORTDISCUSS, and CINAHL.

**Table 2:** Overview of included studies.

Author Year, Country	Aim	Participants	Measurement Method and Operationalisation of β-hydroxybutyrate (β-OHB) Levels and Hyperglycaemia	Results	Conclusion
		Age (Mean ± Standard Deviation (SD)			
		Gender, Diabetic Status			
Tantiwong et al. 2005 [6], Thailand	Comparison of capillary β-OHB measurements with urine ketone testing in diagnosing DKA.	19 DKA (45.6 ± 16.95 years)	Capillary blood ketones meter (MediSense Optium^TM^) β-OHB ≥ 3.0 mmol/L diagnostic of DKA Blood glucose ≥ 250 mg/dL (13.9 mmol/L)	The β-OHB value of >3.0 mmol/L had a sensitivity of 90% and a specificity of 100%, whereas urine ketones had a sensitivity of 95% and a specificity of 100% in diagnosing DKA.	β-OHB and urine ketones are effective to confirm DKA in uncomplicated cases.
		42.1% type 1 diabetes			
		38.8% type 2 diabetes			
		19.1% unclassified type of diabetes			
Naunheim et al. 2006 [23], USA	Comparison of capillary β-OHB measurements with standard measures (arterial blood gases, anion gap and carbon dioxide) for accuracy in predicting DKA in the ED.	160 (41 ± 15 years)	Capillary blood ketones meter (Precision Xtra, Abbott Laboraotries) No pre-operationalisation of β-OHB levels Blood glucose ≥ 250 mg/dL	High correlation between β-OHB levels and anion gap (*r* = 0.66, *p* < 0.001), carbon dioxide (*r* = −0.69, *p* < 0.001), and blood glucose (*r* = 0.31, *p* < 0.001).	A β-OHB test can accurately identify patients with DKA, adding this at triage is likely to decrease delays in recognition of DKA in ED.
		46.8% female			
		103 Non-DKA (38 years)		The β-OHB value of >1.5 mmol/L had a sensitivity of 98% (95% CI: 91–100), a specificity of 85% (95% CI: 78–91), with a positive likelihood ratio of 6.7 (95% CI: 4.22–10.78), and negative likelihood ratio of 0.021 (95% CI: 0.003–0.144) of DKA.	
		57 DKA (38 years)			
		Raised blood glucose			
Charles et al. 2007 [24], Singapore	Comparison of capillary β-OHB measurements with clinical diagnosis, venous bicarbonate levels and urine ketone testing in assessing DKA.	111 (median 60 years)	Capillary blood ketones meter (MediSense Optium^TM^) No pre-operationalisation of β-OHB levels Blood glucose ≥ 14.0 mmol/L	The β-OHB value of 1.5 mmol/L compared with other diagnostic methods of DKA had a sensitivity of 100% (95% CI: 59.0–100), a specificity of 93.3% (95% CI: 86.6–97.2), a positive predictive value of 50% (95% CI: 23.0–77.0), and a negative predictive value of 100% (95% CI: 96.3–100).	β-OHB testing is a simple tool that can support early identification of DKA.
		45.5% female			
		4.5% type 1 diabetes			
				The β-OHB value of 3.5 mmol/L, compared with other diagnostic measures of DKA had a sensitivity of 100% (95% CI: 59.0–100), a specificity of 100% (95% CI: 96.5–100), a positive predictive value of 100% (95% CI: 59.0–100) and a negative predictive value of 100% (95% CI: 96.5–100).	
		95.5% type 2 diabetes			
Taboulet et al. 2007 [14], France	Comparison of capillary β-OHB measurements with urine ketone testing in hyperglycaemic patients in the ED.	529 (53 ± 17 years)	Capillary blood ketones meter (Optium, Abbott Laboratories) β-OHB ≥ 1.0 mmol/L Blood glucose ≥ 250 mg/dL (13.9 mmol/L)	Urine ketones scored as +, ++ and +++ corresponded to medium capillary β-OHB levels of 0.5 mmol/L (IQR: 0.1–0.9), 0.7 mmol/L (IQR: 0.2–1.8) and 3 mmol/L (IQR: 1.4–5.2) respectively.	β-OHB is more accurate than urine ketones to confirm ketoacidosis in the ED.
		36% female			
		Raised blood glucose		The β-OHB value of ≥ 3.0 mmol/L was associated with the relative risk of DKA or hospitalization 74 (95% CI: 44–88) and 2.9 (95% CI: 2.5–3) respectively than ketones scored as +++ ketones 31 (95% CI: 18–45) and 2 (95% CI: 1.7–2.1) respectively.	

				Confirmed evaluation of blood ketones beyond 2.9 mmol/L is associated with higher risk of DKA than +++ urine ketones.	
Voulgari and Tentolouris 2010 [25], Greece	Comparison of serum with capillary β-OHB and urine ketones in detecting DKA in ED.	400 Non-DKA (58.5 ± 9.7 years)	Capillaroy blood ketones (Precision-Xtra device Abbott Laboratories) β-OHB > 1.0 mmol/L Blood glucose >13.9 mmol/L	Capillary and serum β-OHB were highly significantly correlated (*r* = 0.99, *p* < 0.001).	Capillary β-OHB of >3.0 mmol/L offers the best combination of sensitivity and specificity for diagnosis of DKA.
				The capillary β-OHB value of >3.0 mmol/L had a sensitivity of 99.87% and specificity of 92.89% with positive predictive value of 92.89% for the diagnosis of DKA. Urine ketones had a sensitivity of 89.89% and specificity of 52.73% with positive predictive value of 41.87% for the diagnosis of DKA.	
		48% female			
		50 DKA (60.2 ± 8.2 years)			
		48% female			
		Insulin-treated type 2 diabetes			
Arora et al. 2011 [26], USA	Comparison of capillary β-OHB measurement with urine ketone testing in assessment of DKA in the ED.	462 Non-DKA (48, age range 40–57 years)	Capillary blood ketones meter (Precision Xtra, Abbott Laboraotries) β-OHB ≤ 1.5 mmol/L insignificant β-OHB > 1.5 mmol/L raised Blood glucose ≥ 250 mg/dL	Urine dipstick sensitivity of 98.1% (95% CI: 90.1–100), a specificity of 35.1% (30.7–39.6), a positive predictive value of 15% (11.5–19.2) and a negative predictive value of 99.4% (96.6–100) for DKA.	β-OHB is more specific than urine ketone testing, therefore β-OHB testing could significantly reduce unnecessary DKA work-ups in the ED.
		35.3% female			
		44.5% insulin-requiring		The β-OHB value of >1.5 mmol/L had a sensitivity of 98.1% (95% CI: 90.1–100), a specificity of 78.6% (74.5–82.2), a positive predictive value of 34.9% (27.3–43) and a negative predictive value of 99.7% (95% CI: 98.5–100) of DKA.	
		54 DKA (41, age range 30–48 years)			
		27.8% female			
		56.6% insulin requiring			
Kinsella et al. 2012 [9], Australia	The use of capillary β-OHB measurement in early assessment of hyperglycaemia in the Emergency Department (ED)	72 (60.97 ± 22.07 years)	Capillary blood ketones meter (Optium Xceed—Abbott Laboratories, Illinois) β-OHB ≥ 1.0 mmol/L elevated Blood glucose ≥ 10.0 mmol/L	Modest correlation between capillary β-OHB levels and blood glucose levels on presentation (*r* = 0.29, *p* = 0.02) and over the ED stay (*r* = 0.21, *p* = 0.01)	β-OHB testing can support early identification of DKA in the ED.
		56.9% female			
		Insulin-requiring diabetics			
Rashid et al. 2013 [27], Bangladesh	Comparison of capillary β-OHB levels with serum electrolytes, urea and creatinine, plasma glucose, and urine ketone testing in assessing and managing DKA.	121 (39 ± 15 years)	Biosensor method (Medisense) β-OHB ≥ 3.0 mmol/L diagnostic of DKA Blood glucose ≥ 12.0 mmol/L	The relative frequencies of DKA, using urinary ketone and capillary β-OHB were 15.6% and 13.9%. Using capillary β-OHB as the reference method, the sensitivity of urinary ketones were 32.6% and the specificity was 93.7%, the positive predictive value, and negative predictive value of urine ketones against blood ketones were 73.68% and 71.84% respectively.	Urine ketone testing has severe limitations in assessing for DKA in patients with type 2 diabetes, with an error of 25%–30%.
		36% female			
		100% type 2 diabetes			
Coetzee et al. 2015 [28], South Africa	Comparison of capillary β-OHB measurement and the gold-standard manual enzymatic method in assessment of DKA	41 (33, age range 17–52 years)	Capillary blood ketones meter (Optium Xceed, Medisense/Abbott) β-OHB < 1.0 mmol/L insignificant β-OHB ≥ 3.0 mmol/L diagnostic of DKA Blood glucose > 13.9 mmol/L	High correlation between capillary β-OHB levels and the manual enzymatic method (*r* = 0.95)	β-OHB capillary testing is as accurate and precise as the manual enzymatic method in confirming DKA.
		58.5% female		Capillary β-OHB levels when compared to the manual enzymatic method demonstrated a sensitivity of 100% and a specificity of 89.5% for diagnosing DKA and a sensitivity of 100% and a specificity of 87.3% for excluding DKA.	
		70.7% type 1 diabetes			
		29.3% type 2 diabetes			
